# Postprandial effects of dietary protein source on metabolic responses, appetite, and arterial stiffness indices in overweight and obese men: the study protocol for a randomized crossover clinical trial

**DOI:** 10.1186/s13063-023-07374-1

**Published:** 2023-06-19

**Authors:** Zahra Dehnavi, Ali Jafarzadeh Esfehani, Omolbanin Hajhoseini, Hanieh Barghchi, Aliyeh Ghannadzadeh Yazdi, Zahra Khorasanchi, Mahdi Shadnoush, Reza Rezvani, Habibollah Esmaily, Mohammad Safarian

**Affiliations:** 1 Department of Nutrition, School of Medicine, Mashhad University of Medical Sciences, Mashhad, Iran; 2 Student Research Committee, Faculty of Medicine, Mashhad University of Medical Sciences, Mashhad, Iran; 3 Metabolic Syndrome Research Centre, Mashhad University of Medical Sciences, Mashhad, Iran; 4 Department of Clinical Nutrition, Faculty of Nutrition Sciences and Food Technology, National Nutrition and Food Technology Research Institute, Shahid Beheshti University of Medical Sciences, Tehran, Iran; 5 Social Determinants of Health Research Center, Mashhad University of Medical Sciences, Mashhad, Iran; 6 Department of Nutrition, Faculty of Medicine, Ferdowsi University of Mashhad Campus, Azadi Square, Mashhad, Iran

**Keywords:** Animal protein, Plant protein, Energy expenditure, Appetite, Arterial stiffness, Crossover clinical trial

## Abstract

**Background:**

Different dietary protein sources are supposed to have various effects on metabolic responses and arterial stiffness in the postprandial period. This study aims to assess the postprandial effects of dietary protein sources, including animal-based protein (AP) and plant-based protein (PP), as part of a high-protein breakfast on appetite response, energy metabolism, and arterial stiffness in overweight and obese men.

**Methods:**

This acute randomized crossover clinical trial will be conducted at the Persian study research center at Imam Reza Hospital, affiliated with the Mashhad University of Medical Sciences, located in the northeast of Iran. Forty-six healthy overweight, and obese men aged 18–60 years will be enrolled based on the eligibility criteria. The subjects will complete two interventions (high-protein AP and PP meals) with 1 week washout period. The primary outcome will be the acute effect of the two test meals on appetite response, energy metabolism parameters, including resting metabolism rate (RMR), diet-induced thermogenesis (DIT), and substrate oxidation (SO), and arterial stiffness indices, including pulse wave velocity (PWV) and pulse wave analysis (PWA). The secondary outcomes include changes in lipemia, glycemia, and insulinemia.

**Discussion:**

The findings of this study will provide novel insight regarding the acute effects of different protein sources on energy metabolism, appetite, and arterial stiffness as a significant cardiovascular disease (CVD) risk factor. It will help dieticians develop effective and efficient meal plans to improve weight reduction and maintenance in overweight/obese individuals.

**Trial registration:**

Iranian Registry of Clinical Trials; code: IRCT20211230053570N1; registered on February 10, 2022

**Supplementary Information:**

The online version contains supplementary material available at 10.1186/s13063-023-07374-1.

## Background

Overweight/obesity is a significant public health problem [1]. The prevalence of overweight/obesity is rapidly increasing at an alarming rate in most parts of the world and is estimated to affect about 20% of the adult population by 2030 [1]. Complex mechanisms have been shown to lead to obesity; however, it is mainly the result of an imbalance between energy intake and energy expenditure [2, 3]. Several studies on the obesogenic and metabolic effects of dietary macronutrients have shown that dietary macronutrient composition can play a pivotal role in energy metabolism and body weight adjustment [4].

It is well documented that dietary protein increases thermogenesis in the postprandial period after meal ingestion. The reason is that the energy cost of the process of digestion, absorption, and metabolization of the protein (23%) is more than that of fat (3%) and carbohydrate (6%) [5]. In addition to thermogenic effects, protein suppresses appetite and energy intake through various mechanisms [6–8]. Therefore, high-protein diets (≥ 25% energy) are suggested to increase weight loss, maintain muscle mass, and prevent weight regain [9, 10].

Regardless of protein quantity, proteins have unique properties associated with the source of protein, amino acid composition, and digestive and absorption kinetics. So, it is suggested that different dietary protein sources exhibit diverse acute and chronic effects on thermic and metabolic responses and appetite control [11–15].

Some studies have shown that animal-based proteins (APs) have greater satiating effects compared to plant-based proteins (PPs) [6, 13, 16]. This difference can be related to the difference in amino acid composition, digestion and absorption rate, protein structure (which influences the digestion and absorption rate), and postprandial incretin hormone responses to APs and PPs [17].

Moreover, based on the results of acute phase studies, different dietary proteins may differently affect energy metabolism markers, for example, energy expenditure (EE), diet-induced thermogenesis (DIT), and substrate oxidation (SO) [11, 12, 18]. Mikkelsen et al. compared the postprandial effects of pork meat and soy protein on 24-h energy expenditure (EE) and found that AP (pork meat) induced a 2% higher EE than PP (soy) [12].

Different dietary protein sources have also been shown to have various effects on lipid and glucose levels in the postprandial period [19]. This difference may be due to the different insulinotropic impacts of protein sources [19]. Some studies have demonstrated that AP ingestion, as a breakfast meal, leads to less significant postprandial changes in the plasma levels of insulin and glucose [20, 21]. In addition, some studies have shown that consuming a diet enriched with APs leads to lower postprandial lipemia by decreasing triglyceride (TG) or free fatty acids levels compared to PPs [22–24].

Postprandial hyperglycemia [25] and hypertriglyceridemia [26] have emerged as independent risk factors of cardiovascular disease (CVD), promoting interest in the cardiovascular effects of postprandial dietary exposure. It has been shown that postprandial lipemia causes acute changes in vascular endothelial function due to increased oxidative stress, which affects arterial stiffness [27]. Arterial stiffness is a measure of vascular function that is strongly associated with CVD risk [28]. Pulse wave velocity (PWV) is the gold standard method, which measures the propagation velocity of pressure waves (m/s) [29]. A research in this regard indicated that high-fat meals lead to a blood pressure-dependent increase in PWV and a decrease in arterial wave reflection [30]. However, data are scarce regarding the effects of dietary protein source on postprandial arterial stiffness.

### Study rationale

According to the above content, it seems that different dietary protein sources have different postprandial effects on metabolic responses, appetite control, and vascular function. However, minor and inconclusive information is available on the difference in postprandial effects between APs and PPs. Therefore, this study was designed to evaluate the postprandial effects of dietary protein source (AP vs. PP) on the mentioned markers in overweight and obese men. We did not include women in the study due to the potential effect of the menstrual cycle on metabolism. On the other hand, to have a more homogeneous population, we only included overweight and obese men in the study. To the best of our knowledge, this is the first study to assess the postprandial effect of dietary protein source on arterial stiffness.

### Study objectives

#### Primary objectives

The primary objective is to evaluate the postprandial effects of dietary protein source (AP Vs. PP) as part of a high-protein breakfast on appetite response, energy metabolism parameters, including resting metabolism rate (RMR), DIT, and SO, and arterial stiffness indices, including PWV and pulse wave analysis (PWA), in overweight and obese men.

#### Secondary objectives

The secondary objective is to evaluate the effects of dietary protein source (AP vs. PP) on postprandial lipemia, glycemia, and insulinemia in overweight and obese men.

### Trial design

**Fig. 1:**
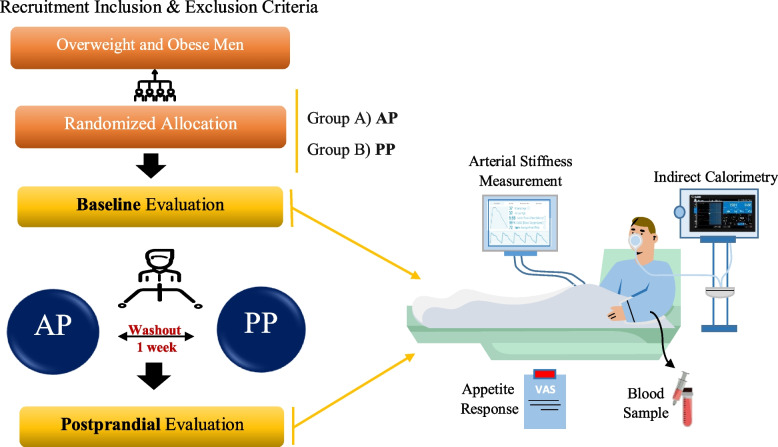
Study diagram of enrollment, interventions, and assessments

This is an acute randomized crossover clinical trial study of two protein-based breakfast meals with different protein sources (AP and PP). Each participant will consume two different test meals on different days with a 1-week washout period in between. Figure 1 shows the diagram of enrollment, intervention, and assessments.

Participants will be instructed not to drink alcohol or engage in severe physical activity within 48 h before each test day. Participants will not be allowed to fast the day before the test days. On the morning of the test day, participants will travel to the research center at Imam Reza Hospital by taxi (with a minimum activity) to arrive at 7 a.m. Participants should abstain from food, drinks (except water), alcohol, and caffeine for 12 h.

On arrival, anthropometric parameters will be measured, and an intravenous catheter will be inserted into an antecubital arm vein. Before consuming the test meals, fasting measurements of indirect calorimetry (IC), PWV and PWA, and subjective appetite will be performed, and venous blood will be collected via the intravenous catheter. Then, participants will be given 15 min to eat the test meals under the researcher’s supervision until the test meal is wholly consumed.

Afterward, postprandial phase tests, including IC, PWA, PWV, and assessing the appetite response, will be performed. Blood samples will also be collected within 6 h. All the tests will be performed in the same conditions (e.g., quiet and temperature-regulated room).

**Fig. 2:**
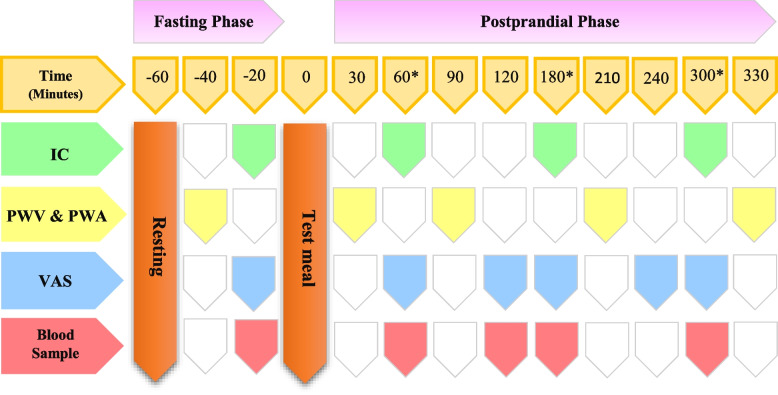
Scheduling study days. Asterisk symbol (*) indicates the following: the exact sequence of measurements at the times points are as follows: (1) blood sampling, (2) appetite assessment, and (3) calorimetry

Figure 2 depicts the schedule of the study days. Time points were determined according to the preliminary results of our previous study. The testing conditions will be repeated on the next test day.

The study protocol was written based on the Standard Protocol Items: Recommendation for Interventional Trials (SPIRIT) Checklist (Table 1).

**Table 1:** Schedule of enrolment, intervention, and assessment based in the Standard Protocol Items Recommendations for Interventional Trials (SPIRIT) guidelines

**Time point**	**Study period**				
	**Enrollment**	**Allocation**	**Post-allocation**		
	**Pre-intervention**	**Time 0**	**baseline**	**Intervention visit 1**	**Intervention visit 2**
**Enrolment**	✓				
Eligibility screening	✓				
Informed consent	✓				
Allocation		✓			
**Interventions**					
AP				✓	✓
PP				✓	✓
**Data collection**					
International physical activity questionnaires		✓			
24-h dietary recall		✓			
Anthropometric parameters		✓			
Body composition		✓			
**IC**					
RMR		✓	✓	✓	✓
SO		✓	✓	✓	✓
DIT		✓	✓	✓	✓
RQ		✓	✓	✓	✓
**Velocimetry**					
cBP		✓	✓	✓	✓
HR		✓	✓	✓	✓
AIx		✓	✓	✓	✓
PWV		✓	✓	✓	✓
PWA		✓	✓	✓	✓
**Laboratory assessment**					
Lipid profile (TG, LDL, HDL, TC, and FFAs)		✓	✓	✓	✓
Serum glucose		✓	✓	✓	✓
Serum insulin		✓	✓	✓	✓

*AP* animal-based protein, *PP* plant-based protein, *IC* indirect calorimetry, *RMR* resting metabolic rate, *SO* substrate oxidation, *DIT* diet-induced thermogenesis, *RQ* respiratory quotient, *cBP* central blood pressure, *HR* heart rate, *AIx* augmentation index, *PWV* pulse wave velocity, *PWA* pulse wave analysis, *TG* triglycerides, *LDL* low-density lipoprotein, *HDL* high-density lipoprotein, *TC* total- cholesterol, *FFA* free fatty acids

## Methods: participants, interventions, and outcomes

### Study setting

This randomized crossover study will be conducted at the Persian study research center at Imam Reza Hospital, affiliated with the Mashhad University of Medical Sciences, located in the North-East of Iran. Participants will be recruited through advertising with posters or email at the Mashhad University of Medical Sciences.

### Eligibility criteria

#### Inclusion criteria

Participants must meet all the following inclusion criteria to participate in this study: men aged 18–60 years old, body mass index (BMI) between 25 and 35 kg/m^2^, being apparently healthy (based on the individual’s self-report and medical history), willing to undertake the required fasting periods, and provision of written consent.

#### Exclusion criteria

The exclusion criteria are as follows: professional athletes; current smoking habits; history of CVDs, hypertension, diabetes mellitus, hyperlipidemia, neurological and/or neuropsychological disorders, and renal disorders, use of medications or supplements that affect energy and protein metabolism (e.g., thyroid medications, supplements containing l-carnitine, ephedrine, caffeine, and antidepressant drugs), use of protein supplements, any supplements for weight loss or weight gain; use of medications or supplements affecting appetite, consumption of toxic substances, history of more than 10% body weigh change within the past 6 months, history of irregular breakfast consumption (less than 5 times a week), having dietary restrictions, fear of getting blood drawn (trypanophobia), inability to partake in the intervention due to intolerance/dietary preferences, and objective diagnosis of hypertension, diabetes mellitus, or hyperlipidemia based on the participant’s biochemical and blood pressure data after they are recruited in the study**.**

### Who will take informed consent?

The eligible participant will be informed by the principal researcher. Written and verbal information regarding the study will be provided to the participants in an easy and understandable language. Then, written consent will be obtained from participants following the trial standard operating processes. Participants will not be included in the study if they decline to give written consent or show substantial distress.

### Additional consent provisions for collection and use of participant data and biological specimens

Contents of the consent form include agreement upon data usage in case of participants’ withdrawal from the study and permission for sharing relevant data with specific academic experts from the universities collaborating in the trial or with other regulatory authorities (Ethic number: IR.MUMS.MEDICAL.REC.1400.399).

### Interventions

#### Explanation for the choice of comparators

In the present study, we aimed to compare two intervention conditions, including AP and PP conditions, and there is no control condition.

#### Intervention description

Intervention condition A: participants will receive an AP test meal. The test meal will provide 20% of the calculated total daily energy requirements and will consist of 30% protein (from animal sources), 40% carbohydrate, and 30% fat.

Intervention condition B: participants will receive a PP test meal. The test meal will provide 20% of the calculated total daily energy requirements and will consist of 30% protein (from plant sources), 40% carbohydrate, and 30% fat.

To estimate the total daily energy requirements of the subjects, the Harris–Benedict Eq. [31] will be used as follows:

1. For women—BMR = 655.1 + (9.563 × weight in kg) + (1.850 × height in cm) − (4.676 × age).
2. For men—BMR = 66.5 + (13.75 × weight in kg) + (5.003 × height in cm) − (6.75 × age).

After calculating BMR by the above formula, TEF and physical activity will be added to calculate TEE.

The test meals will be prepared in the Nutrition Department kitchen at the Mashhad University of Medical Sciences, Mashhad, Iran. The composition of the test meals is presented in Table 2. Two test meals will be isocaloric and similar in macronutrient and fiber content. The palatability of the test meals will be measured using the visual analog scale (VAS). The viscosity of the meals will be measured using a Brookfield Synchro-Lectric Viscometer (Brookfield Engineering Laboratories, Inc.).

**Table 2:** Ingredient composition of the test meals

	**AP**	**PP**
**Protein source**	Chicken breast, egg, yogurt	Soy protein
**Other ingredients**	White toast, potato, carrot, pickled cucumber, parsley, sunflower oil	White toast, mushroom, bell pepper, onion, tomato paste, sunflower oil

*AP* animal-based protein, *PP* plant-based protein

#### Criteria for discontinuing or modifying allocated interventions

No particular side effects are expected following the test meals in the present study. The possible reasons for stopping the intervention will be the occurrence of any intervention or measurements-related side effects. The Ethics committee of Mashhad University of Medical Sciences (MUMS) will decide on the referred cases.

#### Strategies to improve adherence to interventions

The strategies considered to improve the adherence of the participants to the intervention include; preparing meals in accordance with Iranian culture and taste, measuring the palatability of the test meals using VAS, and careful monitoring of participants during the intake of test meals.

#### Relevant concomitant care permitted or prohibited during the trial

This trial includes no relevant concomitant care.

#### Provisions for post-trial care

A physician and nurse will be present, and the participants’ symptoms will be checked during the project (6 to 8 h). After removing the catheter, blood pressure, puncture site bleeding, and other symptoms, such as dizziness, will be checked.

### Outcomes

The primary outcome of this trial will be the comparison of the acute effects of the two test meals on appetite response, and energy metabolism parameters, including resting energy expenditure (REE), DIT, and SO, and arterial stiffness indices (PWV and PWA) in overweight and obese men.

The secondary outcome is to compare the effects of two test meals on postprandial lipemia, including free fatty acids (FFA), low-density lipoprotein (LDL), high-density lipoprotein (HDL), total cholesterol (TC), and triglycerides (TG), glycemia (blood glucose), and insulinemia (serum insulin) in overweight and obese men.

### Participant timeline

The time schedule of enrolment, interventions, and assessments is shown in Table 1.

### Sample size

The sample size was calculated concerning the primary outcomes observed in our previous study and considering desired power of 80% and 95% confidence interval. Considering the mean and standard deviation for the difference in REE of 250 and 330 kcal/day, respectively, an average correlation of 0.5, and a possible dropout rate of 10%, the sample size was calculated as 46 participants (23 participants in each group).

$${\varvec{m}}=\frac{{2\left({{\varvec{z}}}_{1-\frac{\boldsymbol{\alpha }}{2}}+{{\varvec{z}}}_{1-{\varvec{\beta}}}\right)}^{2}\boldsymbol{ }{{\varvec{\sigma}}}^{2}[1+{\varvec{n}}(1-{\varvec{\rho}})]}{{{\varvec{n}}{\varvec{d}}}^{2}}\boldsymbol{ }=\boldsymbol{ }\frac{{2\left(1/96+0/84\right)}^{2}\times {330}^{2}\times [1+4(1-0.5)]}{4{<span class='reftype'>(250)</span>}^{2}}=21\simeq 23$$

### Recruitment

Participants will be identified and selected from the staff and students of the Mashhad University of Medical Sciences who volunteer to participate in the study.

### Assignment of interventions: allocation

#### Sequence generation

As the present study is a crossover study, all participants will receive both interventions. In the present study, only the intervention initiation meal will be randomized. Participants will be randomly allocated to AP or PP conditions based on a 1:1 ratio (simple randomization). The randomization will be performed using a random number table. A random sequence will be generated and sealed in envelopes by a person who is not a member of the research team. Each participant will randomly choose an envelope to be allocated to one of the two test meal conditions for the first trial. Then, the participants will receive the other test meal in the next trial after a 1-week washout period.

#### Concealment mechanism

As participants consume both the protein meals, allocation concealment will be done to identify the sequence of test meal intake.

#### Implementation

Generation of the allocation sequence, enrolment of participants, and assignment of participants to interventions will be done by a person who is not a member of the research team.

### Assignment of interventions: blinding

#### Who will be blinded?

Not blinded.

#### Procedure for unbinding if needed

Not blinded.

## Data collection and management

### Plans for assessment and collection of outcomes

#### Indirect calorimetry

RMR (kcal/d), DIT (kcal/d), and SO (g/d) will be measured by IC. To do calorimetry, participants will be asked to lie down in the supine position while awake and immobile; then, air samples will be collected using a mask (MetaLyzer 3B-R3 device), and respiratory gas (O_2_ and CO_2_) exchange will be measured for 20 min in the fasting phase, followed by postprandial measurements 60, 180, and 300 min after consumption of the test meals. The IC will be calibrated before the start of the measures.

The respiratory quotient (RQ) is the ratio of VCO_2_ to VO_2_ (VCO_2_/VO_2_), which reflects the rate of SO. DIT is the production of heat related to SO during energy uptake. DIT varies according to the quantity and type of oxidized substrate (i.e., carbohydrates, proteins, and fat).

IC will be performed in a quiet area at room temperature and mild lighting and require specific conditions (i.e., free of physical activity and psychological stress, fasting state for more than 6 h before the measurement, and resting in the supine position for > 20 min before the calorimetry). During the measurement, the room temperature will be maintained between 23 and 25 °C.

Given that stress has a negative effect on the quality of the calorimetry, the following measures will be taken to minimize the stress level of the participants:

1. Performing the test in a completely quiet room
2. Providing facilities for the participants to rest in the intervals between assessments and at least half an hour resting before the test
3. In case of determining stress and tension in participants, testing will be postponed to another day

#### Pulse wave analysis and pulse wave velocity

An ultrasound evaluation of the carotid artery will be done in the supine position, with the head turned 45° away from the scanning side. PWA and blood pressure measurement will be conducted using the Sphygmocor XCEL device on the right side of each participant. Blood pressure will be measured after at least 15 min of rest on the right upper arm. Moreover, the measurement of augmentation index (AIx), central blood pressure (cBP), and heart rate (HR) will be done according to the manufacturer’s guidelines for pulse wave analysis. As AIx is inherently heart rate dependent, the AIx75, which standardizes AIx to a heart rate of 75 beats per minute, will also be used.

Furthermore, arterial stiffness measurement will be taken using the tonometer at the femoral and carotid arteries, which give carotid-femoral PWV.

PWV will be examined according the sequentially measured electrocardiogram-gated left carotid and radial waveforms (applanation tonometry) using the stepwise method. The travel distance of the pulse wave will be calculated as the distance from the suprasternal notch to the femoral artery and the distance from the suprasternal notch to the carotid artery using a flexible tape measure. The measurements will be performed at least twice, and the mean PWV will be applied to the analysis. PWV will be measured at baseline and 30, 90, 210, and 330 min after the test meals.

#### Appetite response

During the test day, participants will be given a paper-based questionnaire before and every 1 h after the test meal to record their feelings of hunger and satiety. Appetite questions are assessed based on (a) satiety score card, (b) visual analog scale, (c) satiety labeled intensity magnitude scale, (d) unlabeled visual hunger/fullness category scale, and (e) “Teddy the Bear” hunger and satiety rating scale. The calculated score represents the most extreme sensation the participant had experienced and the response options or satiety or motivation-to-eat in a wide range to be well expressed.

#### Blood samples

Blood samples will be collected before and 60, 120, 180, and 300 min after meal consumption to evaluate metabolic markers. A maximum of 2 ml of blood will be collected from each participant at each of the given times (overall 10 ml/day) to measure glucose, insulin, TG, LDL, HDL, TC, and FFAs. The blood samples will be centrifuged immediately, and the serum samples will be stored at − 20 °C. On each test day, measurements will be taken from 7 a.m. till 2 p.m.; we selected this period because it was supposed to represent a typica1 interval between breakfast and lunch.

#### Anthropometric parameters and body composition

The anthropometric parameters will be measured at the beginning of the study by a trained nutritionist. Body weight will be measured on a clinical scale (SECA) to the nearest 0.1 kg. Height will be measured with a wall-mounted stadiometer to the nearest 0.1 cm. Based on the obtained measurements, BMI will be calculated (weight (kg)/height (m^2^). Waist circumference will be measured at the midline between the lower border of the ribs and the iliac crest in a horizontal plane to the nearest 0.5 cm. Finally, the body composition of the participants will be analyzed using bioelectrical impedance analysis (InBody 270-Waynesboro YMCA).

#### Dietary measurements

A 3-day food record will be collected from all participants at baseline. Moreover, dietary intakes will be assessed using a 24-h dietary recall on each test day; the collected data will be analyzed by the nutritionist 4 software.

#### Physical activity

The level of physical activity in the participants will be assessed using the long form (27 items) of the International Physical Activity Questionnaires (IPAQ) that has been validated for the Iranian population [32]. IPAQ examines four domains of physical activity, including work (7 items), transportation (6 items), household (6 items), and leisure time activities (6 items) during the past 7 days. It also has two questions about sitting time.

#### Strategies to promote participant retention and complete follow-up

On the two test days, the researcher will be present at all stages of the study and measurements and will monitor the study process. Phone calls will be arranged to remind participants regarding the next test appointment.

#### Data management

In the current study, the database will be generated from IC, Sphygmocor XCEL, and laboratory assay results in the Excel software and SAS software version 9.4. To increase the accuracy of data collection, all data will be collected by principal investigators. Finally, data will be analyzed using the SAS software. For data security, usernames and passwords will be provided only to the study researchers.

#### Confidentiality

The data of the participants will be stored securely. A coded ID number will be considered to record the laboratory specimens, reports, and collected data. The collected data will remain confidential, and only the research team will have access to the collected data. For international prospective meta-analyses, the anonymized data will be made available to other researchers.

#### Plans for collection, laboratory evaluation, and storage of biological specimens for genetic or molecular analysis in this trial/future use

For future use, the remaining samples will be stored at − 80 °C at Mashhad University of Medical Sciences.

#### Statistical methods for primary and secondary outcomes

Data will be defined as means ± SDs for the normally distributed continuous variables and median and interquartile range for non-normally distributed continuous variables. Qualitative variables will be expressed as the distribution frequency in different categories (%). Two-sample independent *t* tests will be used to analyze comparisons between the conditions. Viscosity and palatability of the test meals will be analyzed using independent *t*-tests.

Statistical analyses will be performed using PROC GLIMMIX in SAS version 9.4. The carryover effect will be tested; the impact of time and protein source will be calculated if the carryover effect is significant; and if not, only the comparison will be done in parallel in the first period as a covariance analysis. The 2-factor repeated-measures design will be analyzed as generalized linear mixed models to assess the effect of time and protein source in various phases of the study.

#### Interim analyses

This study does not consider interim analysis. If side effects occur frequently, the intervention will be stopped, and the report will be submitted to the Ethics Committee of Mashhad University of Medical Sciences (MUMS) for decision-making.

#### Methods for additional analyses (e.g., subgroup analyses)

In this study, subgroup analysis will be performed on participants with overweight (BMI between 25 and 30 kg/m^2^) and obese (BMI > 30 kg/m^2^) subgroups. Subgroup analysis will be performed using binary logistic regression adjusting for BMI.

#### Analytical method to handle protocol non-adherence and any statistical methods to handle missing data

Since this study is performed in the acute phase, if participants do not adhere to the protocol, they will either be excluded from the study or asked to repeat the study on another day. Therefore, all the analyses will be done based on the per-protocol principle.

#### Plans to give access to the full protocol, participant level-data and statistical code

We strive to publish the complete study protocol and results as soon as possible, regardless of the extent or direction of the effect. The corresponding author may provide an anonymized data set and statistical code (Email: Safarianm@mums.ac.ir) on reasonable request.

### Oversight and monitoring

#### Composition of the coordinating center and trial steering committee

The ethics committee and Vice Chancellor of Research and Technology of the Mashhad University of Medical Sciences will supervise and organize all stages of the study. This is an academic committee without conflicting interests.

#### Composition of the data monitoring committee, its role and reporting structure

The Ethics Committee of MUMS will be fully informed from the first version of the protocol to the end of the research process and will control the accuracy of the trials at least twice.

#### Adverse event reporting and harms

Severe adverse events are not anticipated in this study; however, potential minor events may happen, which will be reported to the Ethics Committee of Mashhad University of Medical Sciences (MUMS) for further decisions.

#### Frequency and plans for auditing trial conduct

The study protocol will be evaluated by a team of research ethics professionals, and the procedure of the study will be evaluated by the assigned supervisor when 5% of the study participants are recruited based on the university regulations. Finally, the study procedure will be assessed at the end of the study by the same evaluation team.

#### Plans for communicating important protocol amendments to relevant parties (e.g., trial participants, ethical committees)

Each modification to the protocol that may affect the running of the study will be approved by the ethical committee of MUMS before implementation.

#### Dissemination plans

The procedure of the submission and approval of the reports for dissemination via journal publication will last about 4–6 months, and the results will be disseminated regardless of the direction or magnitude of their effects. Moreover, all the conditions associated with publishing the trial results pertain to the corresponding author, and there are no publication restrictions imposed by the sponsor.

## Discussion

Obesity is a global health issue with a tremendous economic burden [33]. As obesity is mainly due to an imbalance in energy intake and expenditure, identifying ways to reduce energy intake may help in successful weight reduction and weight maintenance strategies. As appetite plays a vital role in energy intake, it is a potential target for dietary interventions to reduce weight. Protein was previously found to reduce appetite, and high-protein diets resulted in a greater weight reduction compared to low-calorie diets with different food compositions [9, 10]. Protein intake from different sources may affect differently on metabolism and appetite [17, 34]. There is inconsistency regarding the effects of various protein sources on appetite and REE. Therefore, identifying the sources of protein that have the maximum impact on REE and appetite may help design dietary interventions for overweight/obese individuals. This study was designed to fill the gap of knowledge in this regard.

The novelty of this study is related to the assessment of the pure effect of a high-protein meal of different sources on short-term REE and appetite. As this study will evaluate the effects in a controlled laboratory setting, the possible significant differences can be attributed to the protein sources.

The findings of this study will provide novel insight regarding the thermogenic effects of different protein sources and their impact on postprandial satiety. The results of this study will help dieticians develop effective and efficient meal plans to improve weight reduction and maintenance in overweight/obese individuals.

Investigating the impact of a high-protein meal with different protein sources on PWV will allow a better understanding of the acute effects of these nutrients on the CVD risk factors, and it may shed light on the effects of long-term dietary lifestyles on the risk of CVD. These data can then be used as a reference for further studies on short-, medium-, and long-term effects of different protein sources on CVD risk factors and the incidence of CVD. Furthermore, the findings of this study may be used to identify the possible confounders (protein source) that might have an acute effect on PWV and to update the standard procedures before performing arterial stiffness measurements.

### Trial status

This trial was registered at clinicaltrials.gov (ID: IRCT20211230053570N1) on February 10, 2022. This is the first version of the protocol, written on 2022–11-12. The trial enrollment has not yet begun and is expected to start in 2 months. Data collection is expected to take about 3 months.

## Supplementary Information

**Additional file 1. **Standard protocol items: recommendation for interventional trials (SPIRIT) 2013 Checklist: Recommended items to address in a clinical trial protocol and related documents.

## Data Availability

The datasets collected and/or analyzed during the present study are not publicly accessible due to ethical concerns, but the corresponding author may provide datasets upon reasonable request.

## Abbreviation

REE: Resting energy expenditure
CVD: Cardiovascular disease
PWV: Pulse wave velocity
PWA: Pulse wave analysis
AP: Animal-based protein
PP: Plant-based protein
DIT: Diet-induced thermogenesis
SO: Substrate oxidation
IC: Indirect calorimetry
VAS: Visual analog score
FFA: Free fatty acids
LDL: Low-density lipoprotein
HDL: High-density lipoprotein
TC: Total cholesterol
TG: Triglycerides
RQ: Respiratory quotient
HR: Heart rate
cBP: Central blood pressure
Aix: Augmentation index
BMI: Body mass index
IPAQ: International Physical Activity Questionnaires
SD: Standard deviation

## References

[1] Kelly T, Yang W, Chen CS, Reynolds K, He J (2008). Global burden of obesity in 2005 and projections to 2030. Int J Obes.

[2] Paddon-Jones D, Westman E, Mattes RD, Wolfe RR, Astrup A, Westerterp-Plantenga M (2008). Protein, weight management, and satiety. Am J Clin Nutr.

[3] Soenen S, Bonomi AG, Lemmens SG, Scholte J, Thijssen MA, Van Berkum F (2012). Relatively high-protein or ‘low-carb’energy-restricted diets for body weight loss and body weight maintenance?. Physiol Behav.

[4] Wali JA, Solon-Biet SM, Freire T, Brandon AE (2021). Macronutrient determinants of obesity, insulin resistance and metabolic health. Biology.

[5] Tappy L, Jéquier E, Acheson K (1993). Thermic effect of infused amino acids in healthy humans and in subjects with insulin resistance. Am J Clin Nutr.

[6] Anderson GH, Moore SE (2004). Dietary proteins in the regulation of food intake and body weight in humans. J Nutr.

[7] Moran LJ, Luscombe-Marsh ND, Noakes M, Wittert GA, Keogh JB, Clifton PM (2005). The satiating effect of dietary protein is unrelated to postprandial ghrelin secretion. J Clin Endocrinol Metab.

[8] Weigle DS, Breen PA, Matthys CC, Callahan HS, Meeuws KE, Burden VR (2005). A high-protein diet induces sustained reductions in appetite, ad libitum caloric intake, and body weight despite compensatory changes in diurnal plasma leptin and ghrelin concentrations. Am J Clin Nutr.

[9] Baba NH, Sawaya S, Torbay N, Habbal Z, Azar S, Hashim S (1999). High protein vs high carbohydrate hypoenergetic diet for the treatment of obese hyperinsulinemic subjects. Int J Obes.

[10] Clifton PM, Keogh JB, Noakes M (2008). Long-term effects of a high-protein weight-loss diet. Am J Clin Nutr.

[11] Acheson KJ, Blondel-Lubrano A, Oguey-Araymon S, Beaumont M, Emady-Azar S, Ammon-Zufferey C (2011). Protein choices targeting thermogenesis and metabolism. Am J Clin Nutr.

[12] Mikkelsen PB, Toubro S, Astrup A (2000). Effect of fat-reduced diets on 24-h energy expenditure: comparisons between animal protein, vegetable protein, and carbohydrate. Am J Clin Nutr.

[13] Diepvens K, Häberer D, Westerterp-Plantenga M (2008). Different proteins and biopeptides differently affect satiety and anorexigenic/orexigenic hormones in healthy humans. Int J Obes.

[14] Gilbert J-A, Bendsen N, Tremblay A, Astrup A (2011). Effect of proteins from different sources on body composition. Nutr Metab Cardiovasc Dis.

[15] Hawley AL, Gbur E, Tacinelli AM, Walker S, Murphy A, Burgess R (2020). The short-term effect of whey compared with pea protein on appetite, food intake, and energy expenditure in young and older men. Curr Dev Nutr.

[16] Gentile CL, Ward E, Holst JJ, Astrup A, Ormsbee MJ, Connelly S (2015). Resistant starch and protein intake enhances fat oxidation and feelings of fullness in lean and overweight/obese women. Nutr J.

[17] Bendtsen LQ, Lorenzen JK, Bendsen NT, Rasmussen C, Astrup A (2013). Effect of dairy proteins on appetite, energy expenditure, body weight, and composition: a review of the evidence from controlled clinical trials. Adv Nutr.

[18] Tan S-Y, Batterham M, Tapsell L (2010). Energy expenditure does not differ, but protein oxidation rates appear lower in meals containing predominantly meat versus soy sources of protein. Obes Facts.

[19] Nilsson M, Stenberg M, Frid AH, Holst JJ, Björck IM (2004). Glycemia and insulinemia in healthy subjects after lactose-equivalent meals of milk and other food proteins: the role of plasma amino acids and incretins. Am J Clin Nutr.

[20] Ratliff J, Leite JO, de Ogburn R, Puglisi MJ, VanHeest J, Fernandez ML (2010). Consuming eggs for breakfast influences plasma glucose and ghrelin, while reducing energy intake during the next 24 hours in adult men. Nutr Res.

[21] Pearce KL, Clifton PM, Noakes M (2011). Egg consumption as part of an energy-restricted high-protein diet improves blood lipid and blood glucose profiles in individuals with type 2 diabetes. Br J Nutr.

[22] Holmer-Jensen J, Mortensen LS, Astrup A, de Vrese M, Holst JJ, Thomsen C (2013). Acute differential effects of dietary protein quality on postprandial lipemia in obese non-diabetic subjects. Nutr Res.

[23] Mortensen LS, Hartvigsen ML, Brader LJ, Astrup A, Schrezenmeir J, Holst JJ (2009). Differential effects of protein quality on postprandial lipemia in response to a fat-rich meal in type 2 diabetes: comparison of whey, casein, gluten, and cod protein. Am J Clin Nutr.

[24] Mamo J, James A, Soares M, Griffiths D, Purcell K, Schwenke J (2005). A low-protein diet exacerbates postprandial chylomicron concentration in moderately dyslipidaemic subjects in comparison to a lean red meat protein-enriched diet. Eur J Clin Nutr.

[25] Cavalot F, Petrelli A, Traversa M, Bonomo K, Fiora E, Conti M (2006). Postprandial blood glucose is a stronger predictor of cardiovascular events than fasting blood glucose in type 2 diabetes mellitus, particularly in women: lessons from the San Luigi Gonzaga Diabetes Study. J Clin Endocrinol Metab.

[26] Bansal S, Buring JE, Rifai N, Mora S, Sacks FM, Ridker PM (2007). Fasting compared with nonfasting triglycerides and risk of cardiovascular events in women. JAMA.

[27] Wallace J, Johnson B, Padilla J, Mather K (2010). Postprandial lipaemia, oxidative stress and endothelial function: a review. Int J Clin Pract.

[28] Laurent S, Cockcroft J, Van Bortel L, Boutouyrie P, Giannattasio C, Hayoz D (2006). Expert consensus document on arterial stiffness: methodological issues and clinical applications. Eur Heart J.

[29] Gurovich AN, Braith RW (2011). Pulse wave analysis and pulse wave velocity techniques: are they ready for the clinic?. Hypertens Res.

[30] Lithander FE, Herlihy LK, Walsh DM, Burke E, Crowley V, Mahmud A (2013). Postprandial effect of dietary fat quantity and quality on arterial stiffness and wave reflection: a randomised controlled trial. Nutr J.

[31] Raymond JL (2020). Morrow K. Krause and Mahan’s Food and the Nutrition Care Process e-book: Elsevier Health Sciences.

[32] Vasheghani-Farahani A, Tahmasbi M, Asheri H, Ashraf H, Nedjat S, Kordi R (2011). The Persian, last 7-day, long form of the International Physical Activity Questionnaire: translation and validation study. Asian J Sports Med.

[33] Blüher M (2019). Obesity: global epidemiology and pathogenesis. Nat Rev Endocrinol.

[34] Veldhorst MA, Nieuwenhuizen AG, Hochstenbach-Waelen A, van Vught AJ, Westerterp KR, Engelen MP (2009). Dose-dependent satiating effect of whey relative to casein or soy. Physiol Behav.

